# Correlation between core stability and the landing kinetics of elite aerial skiing athletes

**DOI:** 10.1038/s41598-023-38435-9

**Published:** 2023-07-11

**Authors:** Ming Wei, Yongzhao Fan, Haiying Ren, Ke Li, Xuesong Niu

**Affiliations:** 1 Shenyang Sport University, Shenyang, 110102 China; 2 Capital University of Physical Education and Sports, Beijing, 100191 China; 3 Comprehensive Key Laboratory of Sports Ability Evaluation and Research of the General Administration of Sport of China, Beijing Key Laboratory of Sports Function Assessment and Technical Analysis, Beijing, 100191 China

**Keywords:** Health care, Quality of life

## Abstract

Core stability is critical for improving athletic performance, reducing injury risks and is one of the most important elements of athletic training. However, the effect of core stability on landing kinetics during aerial skiing remains unclear, making relevant analysis and discussion an urgent issue to address. To enhance core stability training and landing performance aerial athletes, this study proposed a correlation analysis to investigate the effect of core stability on landing kinetics. Previous studies on aerial athletes have overlooked landing kinetics and lacked correlation analyses, leading to unsatisfactory analysis outcomes. The correlation analysis can be integrated with core stability training indices to analyze the effect of core stability on vertical and 360° jump landings. Therefore, this study can provide guidance for core stability training and athletic performance in aerial athletes.

## Introduction

Aerial freestyle skiing (aerials) was listed as an official Olympic event in Lillehammer 1994 winter Olympics. With advances in technical movements, athletes have pushed beyond previous limits. Technical movements for female athletes progressed from bFF, bDFF, and bFDF to bLFF, bLTF, bFFF, and bLDFF. Movements for male athletes evolved from bFFF, bLDFF, and bFDFF to bDFFDF and bFDFDF (Table 1). According to judging criteria from the International Ski Federation (FIS) for aerials, aerial maneuvers account for 70% of the total competition score, while landings make up the remaining 30%. However, landing success is more visually impactful than aerials and determines the final score. Therefore, it is necessary to identify effective training strategies to increase landing success rates.

**Table 1:** Jump code with degree of difficulty.

Jump code	Jump description	Difficulty factors
bFF	Back full-full	3.150
bDFF	Back double full-full	3.525
bFDF	Back full-double full	3.525
bLFF	Back lay-full-full	3.800
bLTF	Back lay-tuck-full	3.500
bFFF	Back full-full-full	4.050
bLDFF	Back lay-double full-full	4.175
bFDFF	Back full-double full-full	4.425
bDFFDF	Back double full-full- double full	5.000
bFDFDF	Back full-double full- double full	5.000

Correlation studies can facilitate an in-depth examination of the relationship between core stability (CS) and landing kinetics, clarify the significance of core stability training (CST), and enhance landing performance. Current research has primarily focused on rehabilitation^1^, strength training^2^, and postural control^3^ of aerial athletes, and few have been conducted on their landing performance^4,5^. These limited studies have predominantly taken a theoretical approach, lacking comprehensive practical analyses.

The purpose of this study is to elucidate the influence of core stability on the landing stability, so as to provide guidance for improving freestyle aerial skiing athletes landing success rate.

## Materials and method

### Participants

A total of 18 aerial athletes (8 males and 10 females) without injuries voluntarily participated in this study (master sportsmen: 11, first grade athletes: 7, training history: 8.36 ± 2.16 years, and training frequency: six times/week). Subject characteristics are summarized in Table 2. All participants signed informed consent forms after given written and verbal information about research purpose, procedure, and underlying risks. All experimental protocols were approved by the Ethics Commission of Shenyang Sports University (No. 20211224S), and all methods were performed in accordance with the Declaration of Helsinki.

**Table 2:** Participants characteristics (mean ± SD; n = 18).

Age (years)	Mass (kg)	Height (cm)
16.44 ± 2.13	57.56 ± 8.11	163.11 ± 6.19

### Procedures

Prior to the measurement, the researcher calibrated the instrument (David spine test system (Model: F110, F120, F130, F150, Finland), Kistler force plate (Model: 9255C, Germany), Vicon Motion System (Model: MX/T-Series, UK), HUR balance lab (Model: 141103432005-1, Finland)).

Before the test, the athletes conducted 15 min of warm-up, which focused on dynamic stretching of the core and lower extremities, running and jumping exercises to prevent athletes from being injured during the test, especially maximum strength and jumping test.

#### Core stability test

This study selected the indicators related to core stability and aerial skills, which could reflect core strength (squats and isometric trunk flexion, extension, lateral bending, and rotation), rapid core strength (10 V-up), core endurance (side bridge, L control, and trunk hyperextension), and balance (single foot triple jump, single leg balance, and single leg balance with eyes closed). These indicators are not only but also to.

#### Landing kinetics test

**Figure 1:**
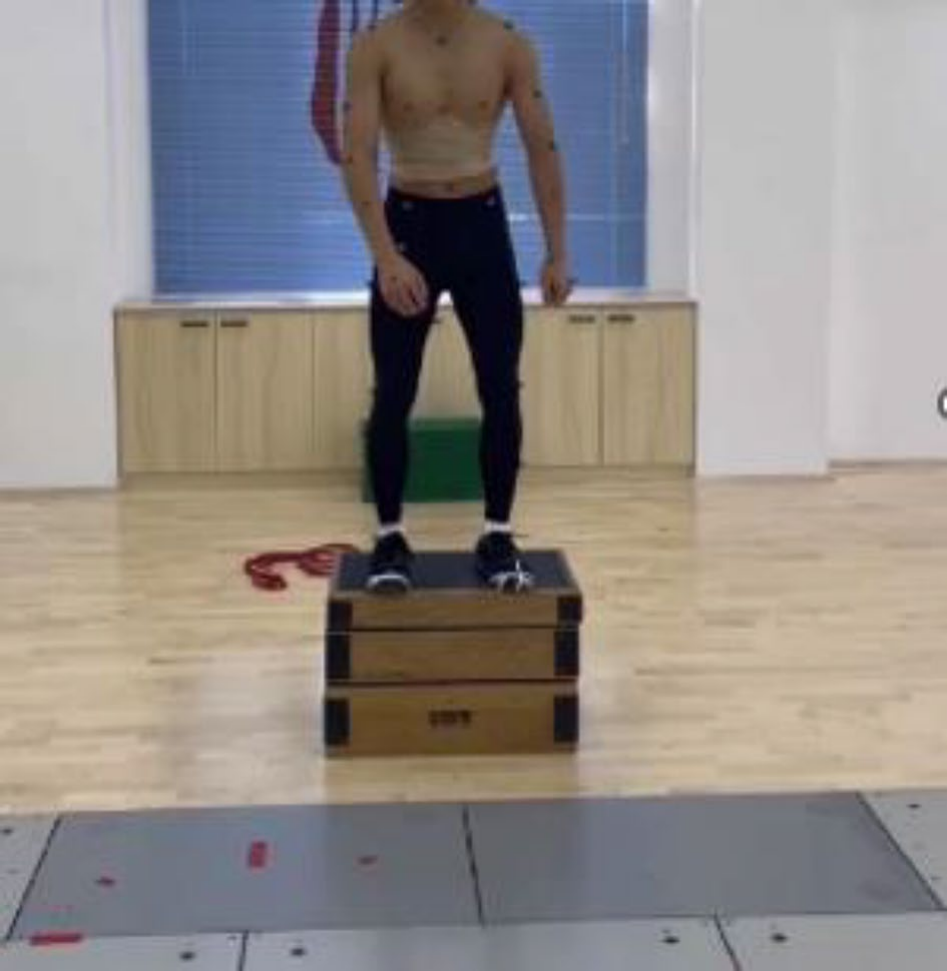
Laboratory of technical diagnosis and skill assessment of the General Administration of Sport of China.

This test was performed in the laboratory of Technical Diagnosis and Skill Assessment of the General Administration of Sport of China (Fig. 1). A Kistler force plate sampling frequency of 1000 Hz and frequency of 100 Hz were used.

**Figure 2:**
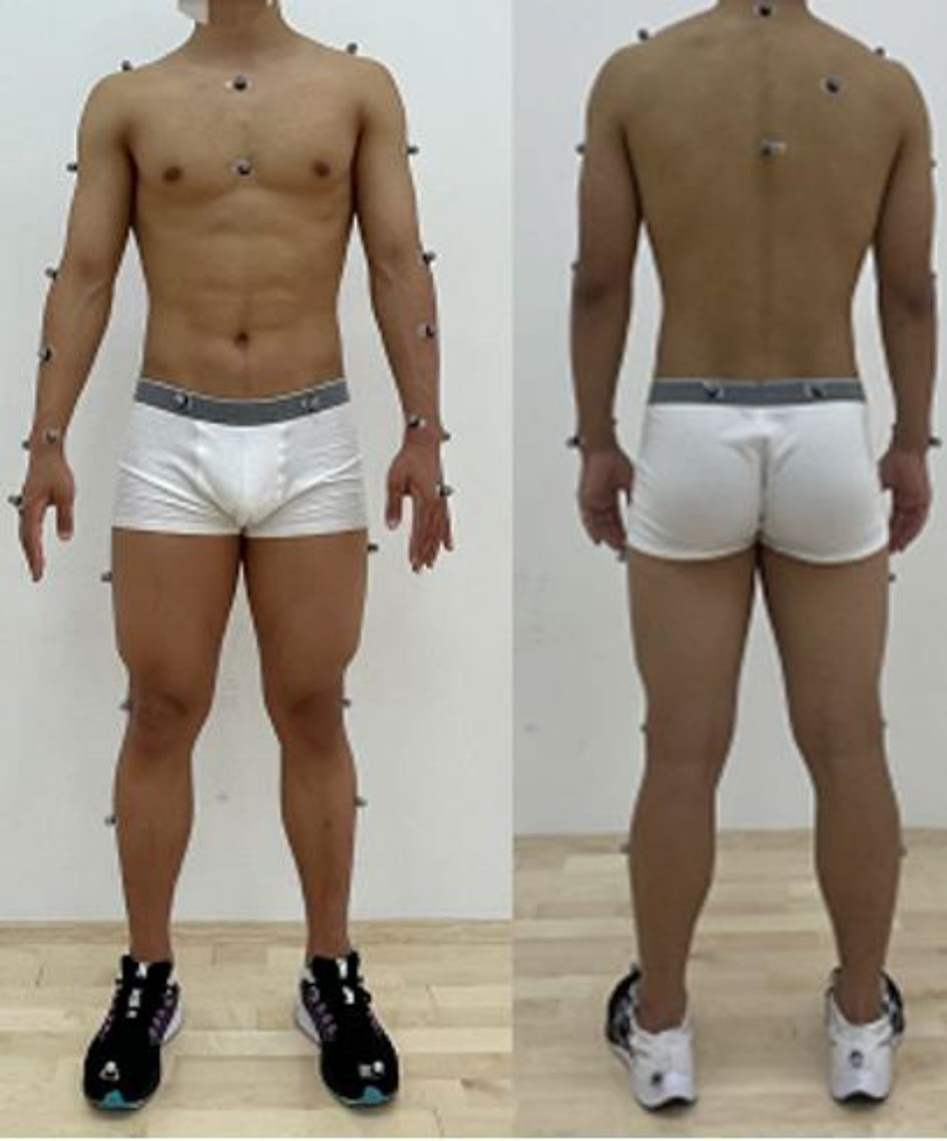
Positions of the marker points.

The marker points were placed on the following body positions: head, C7, T10, sternal stalk, glabella, right scapula, acromion, medial elbow, lateral elbow, forearm, medial wrist, lateral wrist, end of the metacarpal, anterior superior iliac spine, posterior superior iliac spine, thigh, lateral knee, calf, medial ankle, lateral ankle, metatarsal, and heel. To reduce experimental error, the same brand and model of shoes, which only differed in size, were used in this study (Fig. 2)^6^.

The participants were required to conduct a routine of movements in order, respectively, stand on a 50-cm jump box^7^ and remain still for 2–3 s, then squat and jump up, next jump from the jump box to the Kistler force plate, and then complete a vertical jump to the ground, and finally perform a 360° jump to the ground. Each participant performed this routine 3 times each, and sufficient rest (30–60 s) was allowed between the tests.

### Data analysis

SPSS 26.0 software was used for data processing and analysis. Pearson’s correlation analysis was used for normally distributed data, and Spearman’s correlation analysis was used for non-normally distributed data.

## Results and discussion

### Correlation between core stability and landing kinetics

Existing studies on the impact of CST on landing kinetics are contradictory^8^. Brown stated that CST combined with balance training had no effect on knee flexion angle during landing^9^. In contrast, Pfile et al. found that CST reduced knee flexion angle during landing^10^. Sugimoto et al. highlighted that combining strength training with balance training alone could not reduce the risk of ACL injury, whereas CST combined with resistance training had a good preventive effect on ACL injury^11^.

In the study of Tsai^12^, CST was conducted on 16 volleyball players to test the effect of core stability on Lower limb strength. The results showed that the athletes’ hip flexor, hip external rotation, knee flexor, and knee extension strengths increased by 19% (P = 0.001), 14% (P = 0.04), 25% (P = 0.02), and 24% (P = 0.003), respectively. Furthermore, the study found that maximum trunk flexion angle at landing and maximum knee flexion angle decreased by 6.5° (P = 0.03) and 9.5° (P = 0.003), respectively. A biomechanical study showed that a lateral or forward tilt of the trunk facilitated stability during single-leg landings^13^. Defects in neuromuscular control of the core muscle groups could lead to increased trunk displacement during the cushion phase of landing or increased sway during standing^14^. These studies show that CST has a benign effect on landing performance.

**Figure 3:**
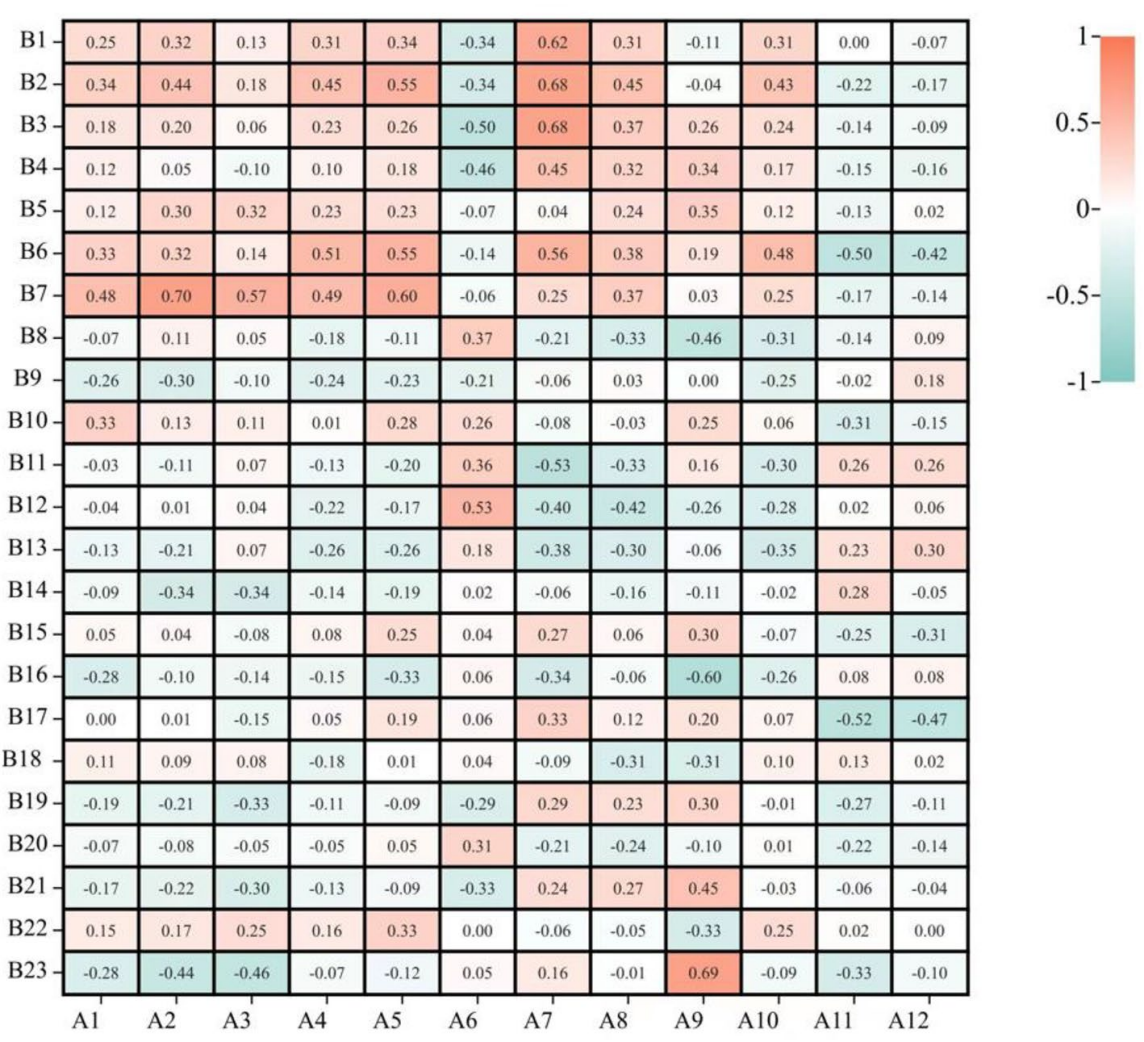
Correlation analysis between core stability and vertical jumping. *Indicates significant correlation, P < 0.05; **indicates highly significant correlation, P < 0.01. *A1* squat, *A2* isometric trunk flexion test, *A3* isometric trunk extension test, *A4* isometric trunk lateral bending test, *A5* isometric trunk rotation test, *A6* 10 V-up, *A7* side bridge, *A8* L control, *A9* trunk hyperextension, *A10* single foot triple jump, *A11* single leg balance, *A12* single leg balance with eyes closed, *B1* first peak time, *B2* first trough time, *B3* second peak time, *B4* second trough time, *B5* total time, *B6* first peak/weight, *B7* first trough/weight, *B8* second peak/weight, *B9* second trough/weight, *B10* first peak/phase, *B11* first trough/phase, *B12* second peak/phase, *B13* second trough/phase, *B14* end/phase, *B15* sagittal axes, *B16* frontal axis, *B17* horizontal axis, *B18* peak power hip range, *B19* peak power hip changing range, *B20* peak power knee range, *B21* peak power knee changing range, *B22* peak power ankle range, *B23* peak power ankle changing range.

In this study, we found that an increase in squat strength during 360° jump landing reduced peak power hip range (r = 0.506, P = 0.032), and single foot triple jump, which reflects neuromuscular control, reduced peak power hip range (r = 0.503, P = 0.034) and peak power knee changing range (r = − 0.495, P = 0.037) (Fig. 3). An increase in core strength would reduce hip flexion angle at landing but increase hip flexion angle when any of core muscles got injured or when a single leg landing was unstable. Stearns^15^ conducted a study on 21 participants who performed hip strength and balance training for 4 weeks to test the biomechanics of their lower extremities during landing and it found that increased hip strength improved landing stability and reduced the risk of ACL injury.

In isometric trunk rotation test, trunk rotation isometric strength was positively correlated with the first trough time of vertical and 360° jumping, thus showing the important role of core anti-rotation ability in stabilizing landing impact to overturn inertia (Fig. 3). Previous studies on spiral myofascial chains have proved that it could lead to and adjust the twisting and rotation of the body and can help the body maintain balance in unstable conditions^16,17^.

**Figure 4:**
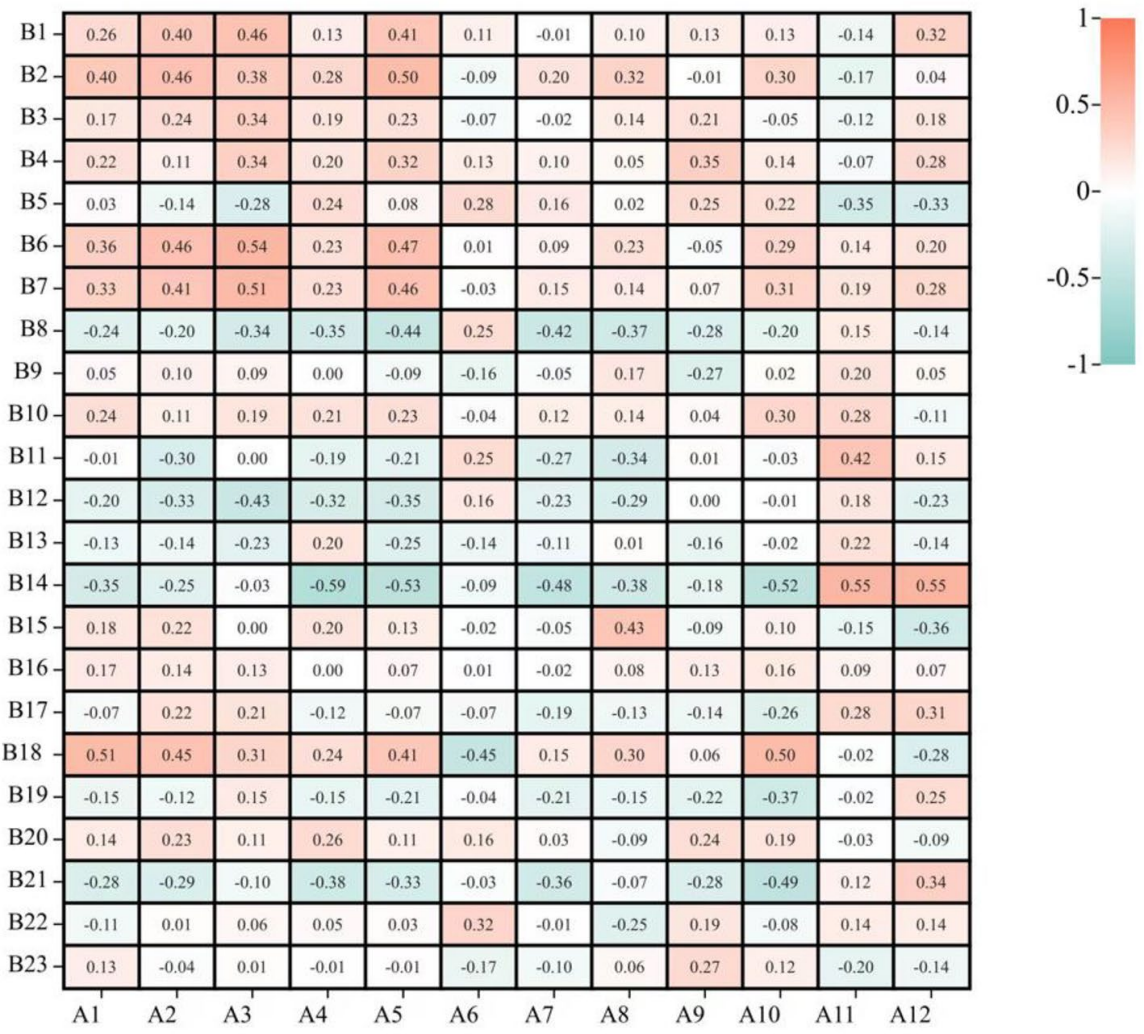
Correlation study between core stability and 360° jumping.

In isometric trunk flexion, extension, lateral bending, and rotation tests, trunk flexion and extension, lateral bending, and rotation were positively correlated with first peak/weight and first trough/weight in both jumping forms (Figs. 3, 4). It means that core strength can buffer greater force and reduce the impact of the second peak and second trough to make landing more stable.

During vertical jumping, 10 V-up was negatively correlated with the second peak time and positively correlated with the second peak/phase, indicating that the rapid concentric contraction of the hip flexor muscles could absorb impact force during the second peak (Fig. 3). Full extension of the lower extremity can resist landing impact in the second trough. It is consistent with the landing kinetics in aerials and gymnastics^18,19^.

During vertical jumping, side bridge was positively correlated with the first peak time, first trough time, second peak time, and first peak/weight and negatively correlated with the first trough/phase. Trunk hyperextension was negatively correlated with frontal axis and positively correlated with peak power ankle changing range (Fig. 3). Improved core endurance prolongs landing time and reduces the impact force per unit time, which can effectively reduce the risk of back and knee injuries^2^. Guo^20^ highlighted that core stability was critical to improving landing stability and body control, which could reduce the risk of knee injury.

During vertical jumping (Fig. 3), single foot triple jump was positively correlated with the first peak/weight, single leg balance was negatively correlated with the first peak/weight and horizontal axis, and single leg balance with eyes closed was negatively correlated with horizontal axis. Balance ability increases core and lower extremity strengths and tends to stabilize after exceeding a specific threshold^21^. Therefore, good balance can help athletes to buffer the impact at first peak, which is conducive to reduce the displacement of the center of pressure in horizontal axis between the peak and trough, thus improving landing stability. Myer^22^ studied dynamic balance training of 11 female athletes. Their results showed that dynamic balance training can improve neuromuscular control and improve landing stability. However, better results will be achieved if it is combined with a plyometric training.

During 360° jumping (Fig. 4), single foot triple jump was positively correlated with peak power hip range and negatively correlated with peak power knee changing range, indicating that good balance could reduce the hip and knee flexion range at landing as an indication of improved landing stability^12^. In addition, end/phase was negatively correlated with single foot triple jump and positively correlated with single leg balance and single leg balance with eyes closed. It showed that good balance could buffer impact in cushion per unit time for human body.

By improving core and lower limb strength, both dynamic and static balance abilities were enhanced and tend to stabilize after a specific threshold^21^. This study found that improving balance ability enabled athletes absorb impact force better during the first peak, which reduced displacement of vertical pressure center between peak and valley, thus improving landing stability.

In addition, the improvement of balance with eyes closed is beneficial for aerial athletes to adapt to outdoor environment (wind and light) as well as landing stability after difficult aerial flips. Hutt^23^ pointed out that closed-eye dancing exercises improved the scores in star balance test for ballet dancers. Furthermore, the exercises helped ballet dancers resist stage lighting, which could be adopted in ballet dancers’ daily classes. Mcnitt^24^ studied the flips of gymnasts and found that the angular velocity of thighs was faster than that of hips during forward flips and slower than that of hips during backward flips, reflecting the limiting difference in visualization by the direction of flip. This present study suggests that aerial athletes should adopt visual interference training to improve landing stability.

## Conclusions

Improving core strength and balance can decrease hip and knee flexion during landing, reduce fluctuations in peaks and troughs along horizontal axis, and counteract inertia of flips after landing. Enhanced rapid concentric contraction of hip flexors optimizes landing technique and quickly absorb the impact at the second peak. Improving core endurance, the key to prevent injury for aerial athletes, can effectively decrease the risk of back and knee injuries (Supplementary Information).

## Supplementary Information

Supplementary Information.

## Data Availability

All data generated and/or analysed during this study are included in this published article [and its supplementary information files].
